# Assessment of the impacts of microbial plant protection products containing *Bacillus thuringiensis* on the survival of adults and larvae of the honeybee (*Apis mellifera*)

**DOI:** 10.1007/s11356-021-12446-3

**Published:** 2021-02-10

**Authors:** Charlotte Steinigeweg, Abdulrahim T. Alkassab, Hannes Beims, Jakob H. Eckert, Dania Richter, Jens Pistorius

**Affiliations:** 1 Institute of Geoecology, Technische Universität Braunschweig, Braunschweig, Germany; 2 Institute for Bee Protection, Julius Kühn-Institut (JKI) - Federal Research Centre for Cultivated Plants, Braunschweig, Germany; 3 Institute for Apiculture, Lower Saxony State Office for Consumer Protection and Food Safety (LAVES), Celle, Germany

**Keywords:** Microbial pest-controlling products, *Bacillus thuringiensis*, Oral toxicity, Honeybees, Exposure, Pollen feeding

## Abstract

This study was aimed at evaluating the effect of a microbial pest-controlling product (MPCP) with the active substance *Bacillus thuringiensis* ssp. *aizawai* (strain: ABTS-1857) on adults and larvae of honeybees. To determine the contamination levels of Bt spores in different matrices, a colony-feeding study under semi-field conditions was performed. Furthermore, two chronic adult trials and a chronic larval study were conducted under laboratory conditions to test the effects of different concentrations of the plant protection product (PPP) on the development and mortality. Possible modifications of the chronic oral toxicity test were assessed by additional pollen feeding. Our results showed that Bt spores were detected in all matrices over the entire test duration in different concentrations, decreasing over time. The survival of adult bees and larvae was negatively affected in laboratory conditions after a chronic exposure to the MPCP depending on the tested concentrations. Moreover, the earliest sign of bee mortality, resulting from exposure to ABTS-1857, was recorded only after 96 h at the highest tested concentration. Pollen feeding to adults significantly increased the survival of the treated bees. In conclusion, the PPP with the Bt strain ABTS-1857 showed an effect on the mortality of adults and larvae under laboratory conditions. Further studies with Bt-based PPPs under realistic field conditions are necessary to evaluate the potential risk of those MPCPs on honeybees.

## Introduction

Recently, increasing numbers of studies have been published regarding the potential adverse effects of chemical plant protection products (PPPs) on insect pollinators including *Apis* and non-*Apis* bees. To overcome the harmful side effects of those PPPs on non-target organisms, biological methods for pest control have been developed. One approach is the use of microbial pest-controlling products (MPCPs), which seem to be highly specific for particular insect orders (Schünemann et al. 2014). During the last decades, a wide range of microbial pest controlling products (MPCPs) has been developed (Köhl et al. 2019). Isolates of entomopathogenic bacteria, such as *Bacillus thuringiensis* (Bt), have been used commercially to control agricultural and forest insect pests (Bravo et al. 2011). Because of the selective impacts on insects of the orders Lepidoptera, Coleoptera, and Diptera as well as the presumed safety of non-target insects (Schnepf et al. 1998), Bt is applied in integrated pest management (IPM) in organic farming and is often combined with beneficial insects, such as pollinators (Dietrich et al. 2014). The toxic action of Bt-based products in the susceptible insects occurs after the uptake in the gut during the sporulation phase of the bacteria, when particularly insecticidal toxins, such as Cry and Cyt toxins, are produced as crystal inclusions (Bravo et al. 2007; Pardo-López et al. 2013).

Despite the presumed selective action, some Cry toxins have been found to be toxic for other insect groups as well (Feitelson et al. 1992; Feitelson 1993; Schnepf et al. 1998). In particular, a Hymenopteran-specific effect has to be excluded because of the common use with different bioagents (Dietrich et al. 2014). During their foraging activity, bees may be exposed to the Bt products either acutely after spray application or chronically by ingestion of contaminated pollen and nectar; thus, the products can be assumed to be transported back to the colony, leading to exposure of in-hive bees and stored matrices. Furthermore, a long-term effect due to the persistence of Bt sprays is possible. Although a fast reduction of Bt population and toxicity in the environment after application is reported (Schnepf et al. 1998; Haddad et al. 2005; Dietrich et al. 2014), the Bt spores still survive several years after use (Addison 1993). However, there is no information about the viability of Bt spores in the collected matrices, i.e., nectar and pollen, in the bee colony after application in different bee-attractive crops.

The results of the few studies investigating the effects of Bt on bees are somewhat inconsistent and vary with the tested strain and concentrations. D’Urso et al. (2017) reported some behavioral symptoms and physiological midgut changes after acute single exposure to *Bacillus thuringiensis* spp. *aizawai* and *kurstaki* (GC-91 strain). Moreover, reduced survival of honeybee workers is observed for further Bt strains (Brighenti et al. 2007; Libardoni et al. 2018; Potrich et al. 2018). However, the toxicity of MPCPs appears to depend on the tested exposure route (Soni and Thakur 2011). In contrast to exposure by feeding, some application methods, such as stripping or spraying of various MPCPs, do not affect the survival of bees under laboratory conditions (Brighenti et al. 2007; Mommaerts et al. 2010; Libardoni et al. 2018; Potrich et al. 2018). Mommaerts et al. (2010) reported 100% worker mortality of tested bumble bees after 1 week of oral exposure at the maximum field recommended rate of *Bacillus thuringiensis* ssp. *aizawai* via sugar water. On the other hand, they did not report any mortality after exposure to *Bacillus thuringiensis* ssp. *kurstaki.*

Several recent studies have focused on the interactions between single species in the microbiome of the bee gut and their community dynamics in relation to the bees’ health (Engel et al. 2016). Motta et al. (2018) showed increased mortality for bees lacking gut microbiota relative to that observed for bees with a conventional gut microbiota when they exposed to the opportunistic bacteria *Serratia marcescens* kz19, which is commonly detected at very low frequencies in the bee gut (Moran et al. 2012). This indicates the role of the gut microbiome as a part of the immune system of bees.

Besides using Bt as plant protection products, some products containing *Bacillus thuringiensis* ssp. *aizawai* (strain: B401) are currently used to control the greater wax moth (*Galleria mellonella* L.) in several countries. However, they are recommended to be applied on the storage wax combs, which can minimize the exposure of bees to the Bt spores. Vandenberg and Shimanuki (1990) reported that honey produced by bees on treated combs placed in the hive contained very low levels of viable *B*. *thuringiensis* spores after 20 weeks. Nevertheless, they did not investigate the effect on bees after inserting the treated frames.

Our aims were (1) to investigate the distribution of the Bt spores in different matrices within the honeybee colony and (2) to assess the effect of a MPCP with the active ingredient *Bacillus thuringiensis* ssp. *aizawai* (strain: ABTS-1857) on adults and larvae of *A*. *mellifera*. To our knowledge, neither the persistence and degradation of Bt in the bee colony nor a chronic larval test under laboratory conditions has ever been investigated before, but the results could be of great importance for bee health. In addition, standardized test systems for the impact of MPCPs on adults and larvae of bees have to be developed to evaluate potential risks for pollinators.

## Materials and methods

### Test organisms

#### *A*. *mellifera*

Honeybee workers and larvae were obtained from healthy colonies at the apiary of the JKI in Braunschweig, Germany. Each colony comprised about 15,000 workers and a fertile 1-year-old queen. One colony with approx. 5000 bees was placed in a tent to investigate the distribution of Bt within the bee colony under semi-field conditions.

#### *B*. *thuringiensis* ssp. *aizawai* (strain: ABTS-1857)

A registered PPP with the active substance *B*. *thuringiensis* ssp. *aizawai* (strain: ABTS-1857) was used. Based on the recommended field application rate, the different concentrations were chosen stepwise to cover environmentally relevant concentrations.

### Experimental procedures

#### Determination of Bt distribution within the bee colony

**Fig. 1:**
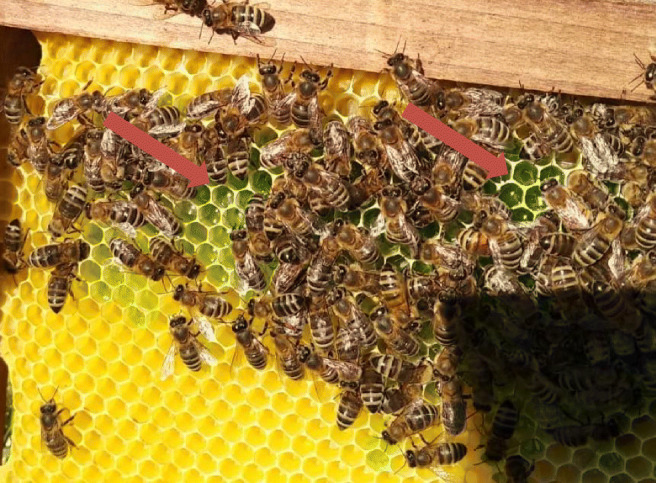
Marked feeding solution using blue food coloring stored in the cells of the treated colony

To investigate the distribution of Bt within the bee colony, a preliminary semi-field feeding experiment with the Bt strain ABTS-1857 was performed in a bee colony. The maximum field recommended rate of the PPP (0.165 %) in orchard was mixed in 2 L of 50% (w/v) sucrose solution and fed to the colony using a feeding bag. The feeding solution was marked by mixing with ca. 2 g of blue food coloring (Dr. Oetker ®) to enable a targeted sampling from the combs (Fig. 1). Samples of nectar, pollen, bee larvae, and adults were taken at regular intervals (days 2, 5, 12, 19) after application.

A sample of approximately 1.0 g was homogenized in an equal amount (w/v) of sterile ddH_2_O. This suspension was used for serial dilution (10^−1^–10^−6^). To detect *B*. *thuringiensis*, 100 μL of each dilution (*n* = 3) was plated on LB agar (10.0 g casein (Roth), 5.0 g yeast extract (Bacto), 5.0 g NaCl (Roth), 15.0 g agar (Gerbu)) and incubated over night at 30 °C.

Phenotypic *B*. *thuringiensis* colonies were counted. Colonies were randomly selected for molecular biological identification as *B*. *thuringiensis* by amplification of the CryIAa region, as described previously (Ogunjimi et al. 2000). Accordingly, a single colony was resuspended in 100 μL sterile ddH_2_O, followed by bacterial lysis at 95 °C for 5 min. After centrifugation at 10,000×*g* for 5 min, 1.0 μL supernatant was used for PCR analysis with OneTaq® DNA Polymerase (NEB) on a ^3^Prime thermal cycler (Techne).

#### Adult bioassays, chronic exposure

Chronic exposure bioassays were carried out according the OECD Guideline no. 245 (OECD 2017). Brood combs with late-stage capped cells were transferred to the laboratory and placed in an incubator under dark conditions (35 ± 2 °C, RH 60 ± 5%) for 48 h until emergence.

Newly emerged workers were collected and caged in groups of ten individuals and maintained in darkness under controlled conditions (33 ± 2 °C, RH 60 ± 5%). Five concentrations of the bioagent varying from 14 to 2730 ppm were daily prepared fresh and mixed in 50% (w/v) sucrose solution. The bees were fed ad libitum with 1-mL syringes, fitted with plugs on the upper side of the cages. The consumed amount per cage, mortality, and behavioral abnormalities were recorded daily and compared to a control (only sucrose solution) and a toxic standard (dimethoate). In the second experiment, half of the bees were exposed to pollen and wax material of the initially used frame in addition to one of three concentrations of the bioagent varying from 14 to 690 ppm. For this, 8 cells containing bee bread were cut out with a scalpel and placed in one corner of the cage, so that the bees could easily reach both matrices.

#### Larval bioassays, repeated exposure

Larval rearing bioassays were conducted according to OECD Guidance no. 239 (OECD 2016) during June and July. In each experiment, the queens of three healthy colonies (replicates) were caged on a wax comb for approx. 24 h to lay eggs. Thereafter, first instar larvae, 24–36 h old, were grafted into 48-welled polystyrene microplates (Greiner Bio-One) lined with Brown Cell Cups. Each cell had prior been prepared with 20 μL of the artificial diet consisting of water, d-glucose, d-fructose, yeast extract, and royal jelly. Subsequently, the microplates were placed in an incubator under dark conditions at 95% R.H. and 35 °C. Two days after grafting (3-day-old larvae), the larvae were fed with 20 μL of a diet exposing them to a dose of the bioagent varying from 0.16 to 32 μg per larvae. The tested concentrations were chosen depending on the maximum detected concentration in nectar in the colony (see Fig. 1). Each group consisted of 16 larvae from each colony (16 × 3; *n* = 48). Larvae were fed over the next 3 days (days 4–6) with 30, 40, and 50 μL diet per individual. The humidity inside the incubator was reduced to ca. 80% on the 7th day after grafting (day 8) and to 50% on the 14th day after grafting (day 15) until emergence. Mortality and morphological differences were recorded when feeding them daily from the 3rd to the 8th day, on the 15th and on the 21st day and compared to a control (only food solution) and a toxic standard (dimethoate). Dead individuals were recorded and removed.

### Statistical analysis

The survival data were analyzed using the Kaplan-Meier test to compare the differences of the survival rates between the control and treated groups over the test duration. The level of significance in all tests was set to ≤ 0.05. All statistical analyses were performed using R (R Core Team (2019)). The packages were “survminer” (Kassambara et al. 2019), “survival,” and “dplyer.” (R: a language and environment for statistical computing; R Foundation for Statistical Computing, Vienna, Austria; https://www.R-project.org/, Version 3.6.1).

## Results

### Distribution of Bt spores within the bee colony

**Fig. 2:**
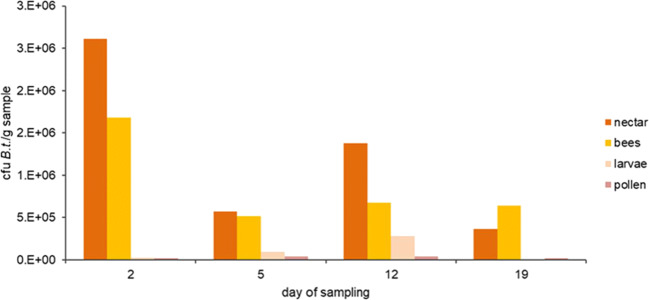
Number of Bt spores in CFU g^−1^ in the sampled matrices nectar, bees, larvae, and pollen over an experimental period of 3 weeks under semi-field conditions

The concentration of the microbial agent *B*. *thuringiensis* ssp. *aizawai*, (strain: ABTS-1857) was quantified in various matrices in the bee colony over a brood cycle period by sampling the matrices nectar, bees, larvae, and pollen. Bt spores were detected in all matrices and were found over the entire sampling period in the tested colony (Fig. 2). The matrices nectar and bees contained the highest levels of Bt spores, whereas pollen and larvae showed lower levels of Bt spores. On the first sampling day, the level of Bt spores was even 85 times higher in nectar than in larvae and pollen. A reduction of the concentration was observed in nectar and bees over the experimental period of 20 days, whereas increasing Bt spore levels were found in larvae.

### Effect of chronic exposure on adult longevity

**Fig. 3:**
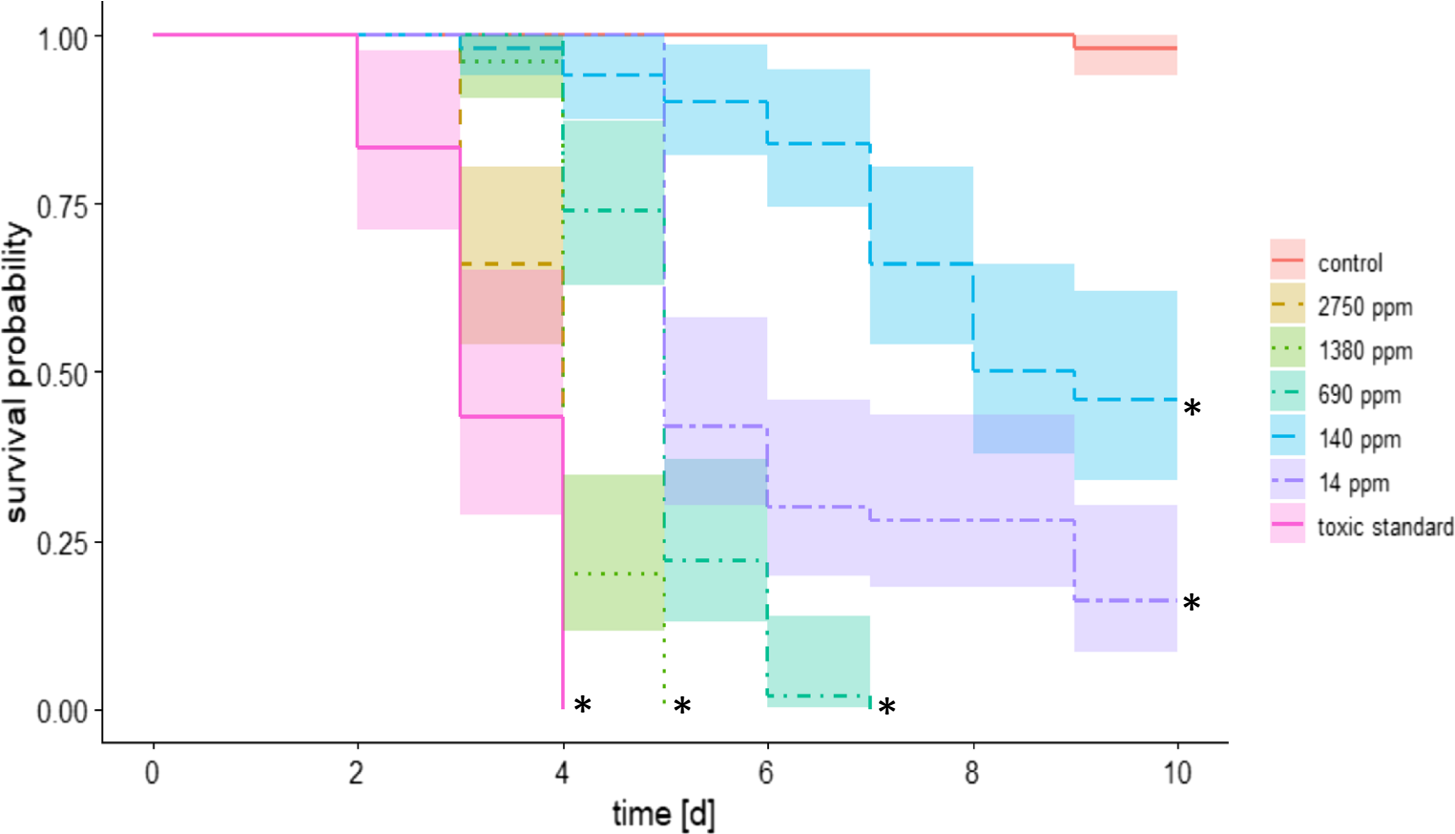
Survival rate of adults, exposed to different concentrations of *the* Bt strain ABTS-1857, compared to the control (C) and the toxic standard (TOX) over the test duration of 10 days (N = 6 cages/treatment, n = 10 bees/cage; Kaplan*-*Meier test; *asterisk* indicates p < 0.05)

In the first experiment, a chronic exposure bioassay was conducted for 10 days to determine the effect of the tested Bt strain on the longevity of adult workers. The survival of adult bees was affected by chronic exposure to the tested product depending on the concentrations (Fig. 3; Kaplan-Meier test, *p* < 0.05). The earliest sign of bee mortality, resulting from exposure to ABTS-1857, was recorded only after 96 h at the highest tested concentration.

**Fig. 4:**
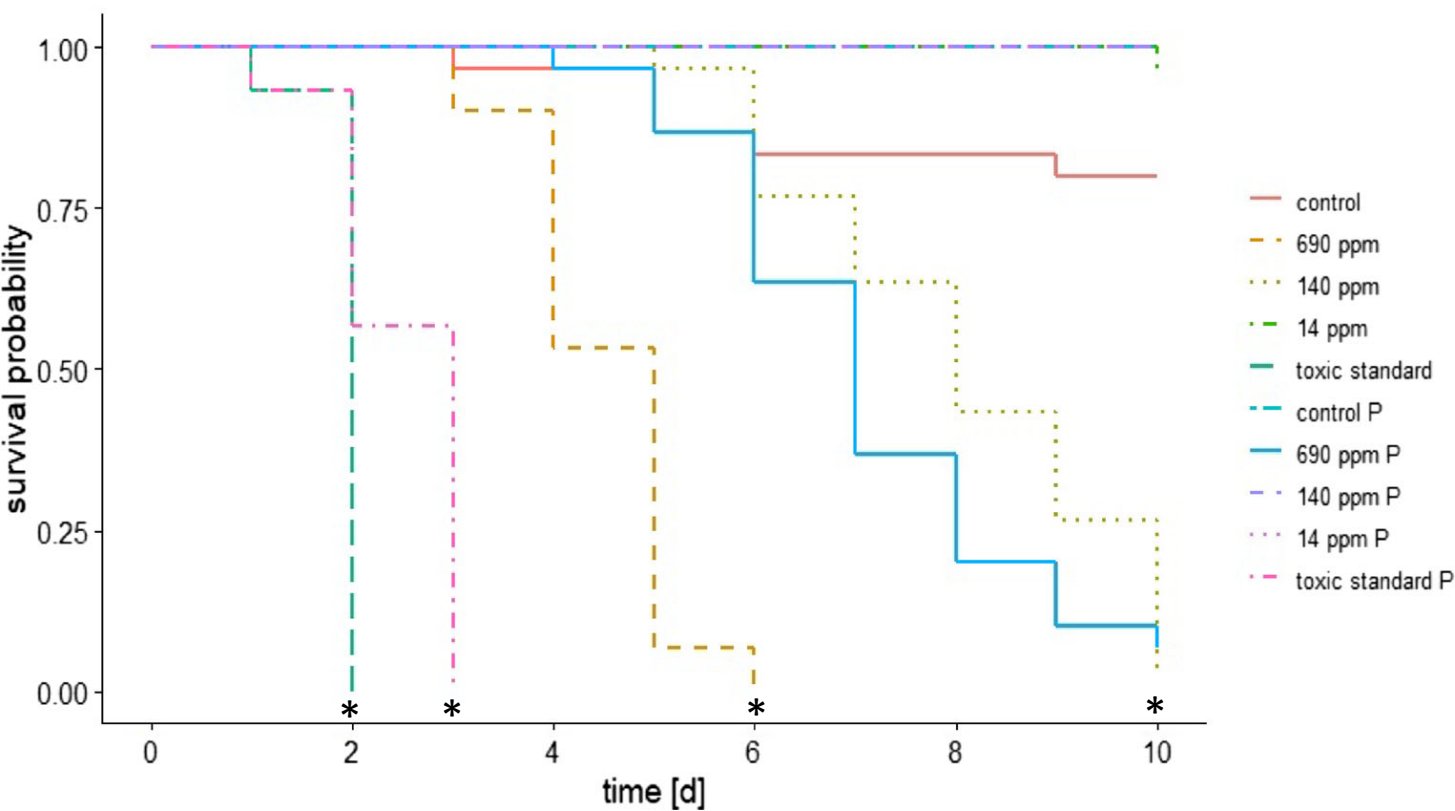
Survival rate of adults, exposed to different concentrations of *the* Bt strain ABTS-1857, compared to the control and the toxic standard over the test duration of 10 days with pollen (labeled as P) and without pollen (N = 3 cages/treatment, n = 10 bees/cage; Kaplan-Meier test; *asterisk* indicates p < 0.05)

In the second experiment, a chronic exposure bioassay was carried out with additional feeding of pollen obtained from the original colony. This additional pollen reduced the sensitivity of bees to Bt compared to bees fed only with sucrose solution (Fig. 4). Bees treated with low concentrations of ABTS-1857 (14 ppm or 140 ppm) survived the experimental period when they were fed pollen, whereas a delaying effect on the mortality was found at the higher concentration (median survival time at 690 ppm, without pollen = 5 days, with pollen = 7 days; at 10 ppm, without pollen = 8 days, with pollen > 10 days; Kaplan-Meier test, *p* < 0.05).

### Effect of chronic exposure on larval longevity

**Fig. 5:**
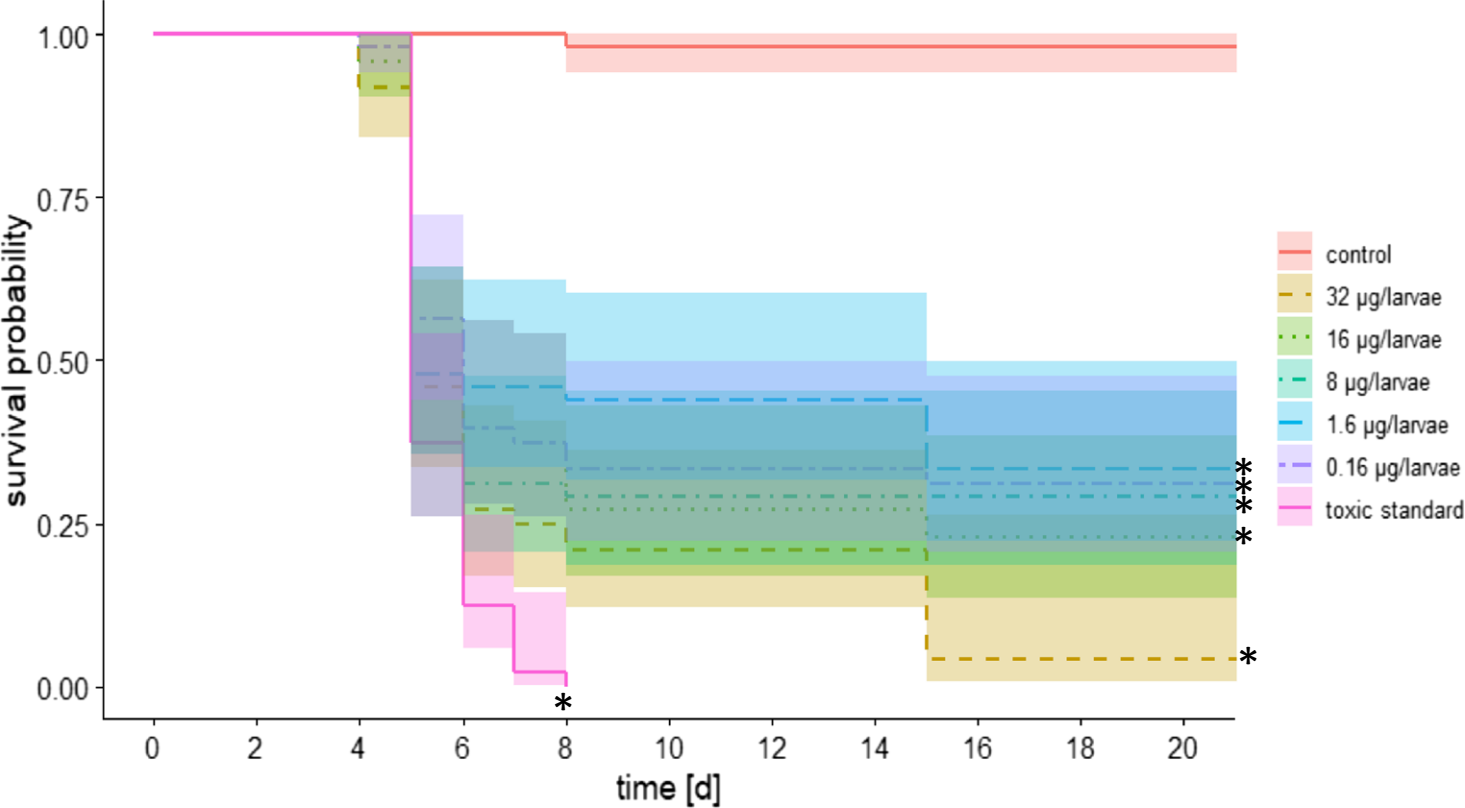
Survival rate of larvae, exposed to different concentrations of *the* Bt strain ABTS-1857, compared to the control and the toxic standard over the test duration of 21 days (N = 1 well plate/treatment, n = 48 larvae/well plate; Kaplan-Meier test; *asterisk* indicates p < 0.05)

To evaluate the impact of the Bt strain ABTS-1857 on the longevity of larvae, a chronic Bt exposure assay was carried out during the larval stage. The development of larvae was recorded until adult emergence (21 days). The survival of larvae exposed to Bt was significantly reduced at all tested concentrations compared to the survival of untreated control larvae (Kaplan-Meier test, *p* < 0.005; Fig. 5). The Bt strain ABTS-1857 had a clear effect on the survival of honeybee larvae, and the major mortality occurred during the larval stages rather than in pupal stages. In all treatments except the highest concentration of the tested product and the toxic standard, pupae were able to develop until adult emergence. The larvae exposed to the toxic reference (dimethoate) showed 100% and the control 0% mortality, validating the test, according to OECD protocol no. 239.

## Discussion

Bt products are generally characterized by a low environmental persistence with a half-life period of viable Bt spores on foliage assumed to be between a few hours and 2 days (Pinnock et al. 1971; Ignoffo and Garcia 1978; Pedersen et al. 1995; Haddad et al. 2005). In contrast, we found Bt spores in all tested matrices of a bee colony at different contamination levels over the entire test period of 3 weeks. A decreasing Bt spore level over time was observed in nectar and bees. Under field conditions, the rapid degradation of Bt spores can be traced to its low UV resistance. Even under controlled greenhouse conditions in terms of temperature, humidity, and UV radiation, a high degree of degradation of Bt spores of the strain ABTS-1857 on leaves and fruits such as tomatoes was measured (Dietrich et al. 2014). Little is known about the behavior and/or vitality of the ubiquitous bacterium under bee colony conditions. Because the conditions differ substantially in the environment and in a bee colony, the persistence and activity of Bt spores depend likely on a different set of parameters, such as the absence of radiation and higher temperature and humidity. Especially temperature and humidity of bee colonies may be limiting factors for the persistence of Bt spores under colony conditions. The toxicity of Bt in orally exposed larvae of *Archips xylosteana* increased with rising temperatures (Li et al. 1995). Thus, temperatures of over 30 °C common in a bee colony may be beneficial for the persistence and activity of Bt. Nevertheless, MPCPs are presumed to be unstable in the colonies’ sector of the worker bee brood (Southwick and Heldmaier 1987) but quite persistent in the sector of the drone brood (Davidson et al. 2003). In contrast to the declining concentration in other matrices, the concentration of Bt in larvae increased over time in our study. This may result from a delayed use of the stored contaminated sugar solution for feeding the larvae when other food resources were absent in the tent. In the first larval stages, the brood is fed by glandular secretions of worker bees. Feeding nectar and pollen only begins at later larval stages (Winston 1987), so the oral intake of Bt via contaminated environmental matrices is expected to occur only for older larvae. Furthermore, the bacteria accumulate due to the periodic feeding of larvae and may thus cause the high mortality when no larvae survived the last sampling day in the tested colony.

The sensitivity of adult worker bees to different MPCP, especially products with Bt, was the focus of various studies (reviewed in Duan et al. 2008). While the majority of the studies showed no effect of Bt, some publications indicated a sensitivity of several bee species to particular Bt products (e.g., Libardoni et al. 2018; Mommaerts et al. 2010; Soni and Thakur 2011). Some strains, such as *B*. *thuringiensis* ssp. *kurstaki* (strain ABTS-351) used in the PPP Dipel®, are proven to be toxic to bees (Malone et al. 1999; Bailey et al. 2005; Brighenti et al. 2007; Mommaerts et al. 2010; Renzi et al. 2016; D’Urso et al. 2017). This indicates that the toxicity for bees depends on the Bt strain (Vandenberg and Shimanuki 1986). Furthermore, Soni and Thakur (2011) showed that the impact of MPCPs on honeybees varies with the exposure route. Bees which were orally exposed to Bt in sucrose solution, had a higher mortality rate than bees exposed by other methods, e.g., spray applications. Libardoni et al. (2018) supported this observation when comparing the effect of exposure routes with three different strains of Bt. In our study, we evaluated a possible effect of the Bt strain ABTS-1857 on adult worker bees with two chronic adult tests, based on OECD Guideline no. 245. The mortality of bees exposed to different concentrations of the PPP with the Bt strain ABTS-1857 was significantly higher than in the control bees, and the effect increased with the concentration. Additionally, the influence of colony material and/or pollen feeding seemed to decrease the sensitivity of adult worker bees when we offered them pollen cells of the original colony in our second chronic adult test. Therefore, one may assume that factors, such as contact with the bee colony and/or food quality and quantity, affect the reactions of bees on Bt. It is known that malnutrition or low food diversity not only lowers immune functions, but may also lead to increased pathogen susceptibility in bees (Alaux et al. 2010; DeGrandi-Hoffman et al. 2010). Accordingly, adequate levels of protein and a variety of pollen increase the immune competence (Alaux et al. 2010).

Under the artificial laboratory conditions in our study, larvae were highly sensitive to different concentrations of the PPP, reaching mortality rates similar to those of the toxic standard. However, larvae which had reached the pupal stage were able to develop normally. Before pupation, larvae defecate, so intestinal contents, such as food remnants or the gut microbiome, are purged (Winston 1987; Kwong and Moran 2016). As a result, accumulated populations of Bt could be removed and rendered incapable of compromising the bees’ development. Also, during the pupation time, larvae are isolated from the colony and do not contact contaminated colony material or food; thus, they are protected from exposure to entomopathogenic organisms. There is some evidence for the toxicity of the strain ABTS-1857 on other pollinator insects; e.g., feeding a PPP to *Bombus terrestris* induced high worker mortality and reduced the reproduction (Mommaerts et al. 2010). Because the loss of brood or young bees can have devastating consequences for a bee colony (Davis 1989), it seems worthwhile to include standardized toxicity tests of Bt strains on honeybee larvae, similar to the one for chemical PPPs reported here, in the registration process.

In view of our results and different studies, the effects of MPCPs on honeybees cannot be extrapolated from one strain to the entire group of a biologically active substance, such as Bt. Moreover, various factors influence the effect of those PPPs, such as UV radiation, humidity, and temperature. It seems essential to evaluate, modify, and adapt the current test systems used for chemical PPPs to reliably measure the potential risk of MPCPs. Adjusting the test duration is one of the necessary modifications. The earliest sign of mortality of the exposed bees under laboratory conditions was recorded only after 96 h at the highest tested concentration, additionally influenced by adult pollen feeding. An acute test with a normal test duration of 48 h or 96 h would not be able to detect these side effects and thus should be considered not sufficient for the registration process. Standardized test guidelines, as established for chemical PPPs, should be developed and applied in the risk assessment of MPCPs. Moreover, exposure routes and different environmental conditions such as the nutrition by pollen feeding appeared to affect the results, too, and have to be taken into account. The mode of action of biological PPPs under the common colony conditions, which vary significantly with the environmental conditions, needs to be investigated. Our study showed the effect of the strain ABTS-1857 on larvae and adult honeybees, but other variables, such as food quality and quantity, strain pathogenicity, weather, or race characteristics, may only be considered by examining natural interactions under field conditions (Duan et al. 2008; Soni and Thakur 2011). The most recent studies on the role of the gut microbiome of honeybees on bee health guide toward further studies determining the impact of MPCPs on the composition and development of the gut microbiome of honeybees. Thorough examinations of the effect of Bt and other MPCPs on the honeybee and other bee species under field conditions will help to understand the natural role and the behavior of “living active ingredients” for beneficial organisms.

## Data Availability

The datasets used and/or analyzed during the current study are available from the corresponding author on reasonable request.
